# Poor linkages in maternal health care services—evidence on antenatal care and institutional delivery from a community-based longitudinal study in Tigray region, Ethiopia

**DOI:** 10.1186/s12884-014-0418-7

**Published:** 2014-12-19

**Authors:** Yohannes Adama Melaku, Berhe Weldearegawi, Fisaha Haile Tesfay, Semaw Ferede Abera, Loko Abraham, Alemseged Aregay, Yemane Ashebir, Friehiwot Eshetu, Ashenafi Haile, Yihunie Lakew, John Kinsman

**Affiliations:** Department of Public Health, Mekelle University, College of Health Sciences, P.O. Box 1871, Mekelle, Ethiopia; Department of Medicine, Mekelle University, College of Health Sciences, Mekelle, Ethiopia; Department of Nursing, Mekelle University, College of Health Sciences, Mekelle, Ethiopia; Center for Disease Control and Prevention, CDC-Ethiopia, Addis Ababa, Ethiopia; Ethiopian Public Health Association (EPHA), Addis Ababa, Ethiopia; Department of Public Health and Clinical Medicine, Umeå Centre for Global Health Research, Umeå University, Umeå, 901 85 Sweden

**Keywords:** Antenatal care, Institutional delivery, Health and demographic surveillance system, Kilte-Awlaelo, Northern Ethiopia

## Abstract

**Background:**

Progress towards attaining the maternal mortality and maternal health targets set by Millennium Development Goal 5 has been slow in most African countries. Assessing antenatal care and institutional delivery service utilization and their determinants is an important step towards improving maternal health care services.

**Methods:**

Data were drawn from the longitudinal database of Kilite-Awlaelo Health and Demographic Surveillance System. A total of 2361 mothers who were pregnant and who gave birth between September 2009 and August 2013 were included in the analysis. Potential variables to explain antenatal care and institutional delivery service utilization were extracted, and descriptive statistics and logistic regression were used to determine the magnitude of maternal health care service utilization and associated factors, respectively.

**Results:**

More than three-quarters, 76% [95% *CI: 74.8%-78.2%*] (n = 1806), of mothers had undergone at least one antenatal care visit during their previous pregnancy. However, only 27% [95% *CI: 25.3%-28.9%*] (n = 639) of mothers gave birth at a health institution. Older mothers, urban residents, mothers with higher education attainment, and farmer mothers were more likely to use antenatal care. Institutional delivery services were more likely to be used among older mothers, urban residents, women with secondary education, mothers who visited antenatal care, and mothers with lower parity.

**Conclusions:**

Despite a relatively high proportion of mothers attending antenatal care services at least once, we found low levels of institutional delivery service utilization. Health service providers in Kilite-Awlaelo should be particularly vigilant regarding the additional maternal health needs of rural and less educated women with high parity.

## Background

Globally, 293,000 maternal deaths occurred in 2013, of which over 99% occurred in low and middle-income countries. In spite of considerable efforts towards attaining the fifth Millennium Development Goal (MDG 5) – which established a global goal of reducing the maternal mortality ratio (the number of maternal deaths per 100,000 live births) by 75% between 1990 and 2015 – maternal mortality has remain stubbornly high throughout much of sub-Saharan Africa [1]. In the southern African region, the maternal mortality ratio has actually increased since 1990, by an annual rate of 2.7%, primarily because of HIV [1]. Other regions have shown modest falls, with rates in West Africa going down by 0.1% per year; by 1.1% per year in Central Africa; and 1.2% per year in East Africa [1]. Consequently, MDG 5 will be achieved in only a very small handful of countries in sub-Saharan Africa.

The situation in Ethiopia reflects that seen in many other countries throughout the region. The overall fall in the maternal mortality ratio of 1.6% per year between 1990 and 2013 has not occurred evenly over this period, with annual falls averaging just 0.6% during the 1990s, and increasing to 2.8% between 2003 and 2013 [1]. Thus, the situation is improving steadily. However, the number of maternal deaths annually remains unacceptably high, with over 15,000 Ethiopian women dying in childbirth in 2013 alone, by far the highest number of any country in the region [1].

Addressing this situation requires a substantial improvement in the coverage and quality of maternal health care services in the country. High quality Antenatal Care (ANC) and skilled attendance during delivery are known to play a significant role in reducing maternal deaths [2-8], and it is critically important that pregnant women utilize both of these services. Antenatal care can reduce the risk of maternal death by eclampsia, through measuring blood pressure, identifying women at risk of eclamptic convulsions, and taking measures to reduce blood pressure whenever possible. Tetanus immunization during pregnancy can also be life-saving for both mother and infant, while the prevention and treatment of malaria among pregnant women, the management of anaemia during pregnancy, and the treatment of sexually transmitted infections (STIs) can all significantly improve fetal outcomes [9].

However, even the best ANC cannot prevent the main causes of maternal deaths that result from complications arising during labour, delivery, and the immediate postpartum period. Around 75% of all maternal deaths take place after the onset of labour [1], and these deaths are caused, in particular, by haemorrhage, hypertensive disorders, and sepsis [10].

Comparison of the findings from studies that investigate utilization of maternal health care services in a number of low and middle-income countries indicates that women are more likely to attend ANC than they are to deliver in health facilities. For instance, in Ethiopia, overall maternal health care service utilization is low, with the 2014 Ethiopian Mini-Health and Demographic Survey (EMDHS) reporting that just 40% of pregnant women made one or more ANC visits, and 32% making the recommended four visits [11]. According to the 2014 EMDHS, institutional delivery took place in only 15% of births [11].

Factors associated with maternal health service utilization have been identified in a variety of African and Asian countries, and they include age of the mother, parity, education level, physical distance to health facility, economic status, and cultural context [12-22]. Furthermore, lack of infrastructure, drugs and supplies at health facilities have also been shown to act as barriers to utilization of institutional delivery [23]. Importantly, however, several studies, including in Ethiopia, have indicated that women who made one or more ANC visit were in turn more likely to seek skilled delivery care [13,14,24-26]. Thus, bringing women into ANC offers the opportunity for increasing their chances of having an institutional delivery, thereby significantly reducing their chance of death during or shortly after childbirth.

This background discussion has included a number of studies that have aimed to assess the magnitude and predictors of ANC and place of delivery in low and middle-income countries, as well as the relationship between the two; but these studies have tended to be based on cross-sectional study designs, and in many cases have therefore been subjected to long recall periods [13,24]. Evidence from population-based longitudinal studies on ANC and institutional delivery services utilization is limited, so this paper investigates the issue using community-based, longitudinal data from the Kilite-Awlaelo Health and Demographic Surveillance System (KA-HDSS) in Tigray region, Ethiopia.

The objective of the study was to assess ANC and institutional delivery service utilization and associated factors in this part of northern Ethiopia, while also exploring the patterns and links between ANC and institutional delivery.

## Methods

### Study setting and period

KA-HDSS was established in April 2009. It consists of 10 “kebelles” (a kebelle is the lowest administrative unit in Ethiopia, with an average of 5000–6000 people), selected from three districts (Kilite-Awlaelo, Wukro, and Atsebi-Wonberta) of Tigray region, Ethiopia. Nine of these kebelles are found in rural areas. In 2011, KA-HDSS became a full member of the International Network of Demographic Evaluation of Populations and Their Health (INDEPTH), a network of 44 HDSSs from the Global South. Detailed descriptions of the KA-HDSS data collectionsystem, data quality control, the database, and the study setting (including a map of the study area) are described elsewhere [27,28].

This study is based on community–based longitudinal data collected by KA-HDSS. We extracted and used data collected as part of KA-HDSS’s ongoing surveillance, concerned with maternal health care service utilization from September 2009 to August 2012, a period of three full years.

### Registration of pregnancies and deliveries

KA-HDSS’s community surveillance includes house-to-house visits that contribute to an ongoing and continuous population events registration of, among other things, pregnancy (including outcome), ANC attendance, and place of delivery. Specially designed pregnancy and delivery (birth) update forms are used, which also include variables that describe maternal socio-demographic and reproductive health characteristics.

Trained fieldworkers identified and registered all pregnancies, deliveries and deaths in the study area during their routine household visits. To ensure timely capture of all demographic events, field workers visit households where such events have occurred every month. These visits are performed in addition to the routine, six monthly updates of all individuals in every household and location within the HDSS catchment area. Each registered pregnancy is monitored until completion and then categorized as resulting in a live birth or stillbirth, an abortion, or a migration if the pregnant woman moved out of the study area.

A total of 2361 women in the reproductive age group (15–49 years), who had complete follow up under continuous observation for their pregnancy and delivery between September 2009 and August 2012, were included in this analysis.

### Outcome measurements

Different studies take different numbers of ANC visits as being optimal. The World Health Organization (WHO) recommends at least four ANC visits during pregnancy, with the first visit taking place within the first trimester [29]. By contrast, some researchers consider two ANC visits to be sufficient [12].

Thus, standardization of the definition of ANC utilization is needed in order to facilitate a comparison of different studies. On this basis, we defined ANC utilization as a mother who had at least one ANC visit, in order to reflect what has been commonly used by the Ethiopian statistics agency in national health and demographic reports [11,24,30].

Deliveries that took place in health institutions and that were assisted by health professionals (doctors, health officers, nurses, midwives, or health assistants) were defined as institutional deliveries; while deliveries that took place at home or in other places were termed as home (non-institutional) deliveries, regardless of who attended the birth [31].

### Predictor variables

The age of the woman at birth, place of residence, educational level, maternal occupation, marital status, and birth order were included as predictor variables in the study. No variables indicating wealth status exist in the KA-HDSS database.

Mother’s age at the birth of her most recent child was categorized into seven sub-categories of five years each (15–19; 20–24; 25–29; 30–34; 35–39; 40–44; and 45–49 years). The educational level of women was defined using years of schooling, and they were grouped into illiterate, primary school, secondary school, and tertiary levels of education. The religion of mothers was categorized as Orthodox Christians and other (Muslim, Protestant and Catholic). Marital status was classified as married, single and dissolved (either widowed or divorced). To characterize mothers by their obstetrics history and other variables, we have included previous pregnancy outcomes, utilization of insecticide treated bed net, and reported history of HIV test. HIV test results are not recorded in the surveillance data.

### Data management and analysis

The KA-HDSS uses the Household Registration System (HRS version 2.1) database, which was customized from other HDSS sites in Africa and Ethiopia. Variables were extracted from the databases and exported to a readable database of Stata version 11.1(Stata Corporation, College Station, TX, USA). For the current analysis, we have included mothers who had complete follow-up records during pregnancy, and who completed their pregnancy within the study period. Descriptive analysis, including proportions, medians, and Inter Quartile Ranges (IQR), were performed. Association between outcome and predictor variables was calculated using odds ratio at p-value <0.05 and 95% confidence interval. Findings were presented in text, tables and graphs.

The association between the outcome variables (antenatal care service utilization and place of delivery) and several predictor variables including maternal socio-demographic characteristics was first analyzed in the bivariate logistic regression model with each independent variable separately. Predictor variables with p-value < 0.05 in the bivariate analysis were retained in the final multivariate logistic regression model. In the multivariate analysis, p-value < 0.05 was considered as a cut-off point for a variable to be considered as an independent predictor of the respective outcome variables. All p-values were computed using Z test.

### Ethics statement

The KA-HDSS received ethical clearance from the Ethiopian Science and Technology Agency (ESTA) with identification number IERC 0030. This study has used pre-existing data from the KA-HDSS which had already received ethical clearance. To capture the occurrence of vital events relating to any family member, the head of a family, an eligible adult, was interviewed. Informed verbal consent was also obtained from the head of the family for all the surveillance data collected. In addition, for specific events like pregnancy and its outcome, the informant was requested to give informed oral consent. This consent procedure was approved by the ESTA. The analysis was based on an anonymous, public-use dataset with no identifiable information on the study participants.

## Results

### Maternal socio-demographic characteristics

Data were analyzed from 2,361 women who had been pregnant and for whom there were records of the outcome. Data for an additional 42 women were excluded due to missing information. Of those women included, the vast majority were residents of rural areas (92.9%; n = 2193). The majority, 73.7% (n = 1741), 80.8% (n = 1907) and 72.8% (n = 1718), were illiterate, married, and housewives, respectively (Table 1).

**Table 1:** **Percentage distribution by background characteristic for reproductive women in age group of 15–49, northern Ethiopia, 2009 to 2012**

**Characteristics**	**Categories**	**n**	**%**
**Age at delivery in years**	15-19	121	5.1
	20-24	493	20.9
	25-29	566	24.0
	30-34	629	26.6
	35-39	388	16.4
	40-44	136	5.8
	45-49	28	1.2
**Median ± IQR**		30 (24, 34)	
**Residence**	Urban	168	7.1
	Rural	2,193	92.9
**Ethnicity**	Tigray	2,347	99.4
	Other	14	0.6
**Religion**	Orthodox	2,342	99.2
	Other	19	0.8
**Maternal educational level**	No formal education	1,741	73.7
	Primary education	452	19.1
	Secondary education	143	6.1
	Above secondary education	25	1.1
**Marital status**	Married	1,907	80.8
	Single	319	13.5
	Divorced	117	5.0
	Widowed	18	0.8
**Maternal occupation**	Farmer	175	7.4
	Daily laborer	134	5.7
	Housewife	1,718	72.8
	Student	139	5.9
	Other	195	8.3
**Relation to head**	Household head	210	8.9
	Housewife	1,784	75.6
	Daughter	320	13.6
	Other	47	2.0

### Respondents’ obstetrics history and maternal health care service utilization

Twelve percent (n = 281) of birth outcomes were from the women’s first pregnancies. There were 1.9% (n = 44) twin pregnancy outcomes and 0.2% (n = 4) abortions or still births during the observation time. The median number of children per women was 4, with nearly three-fifths (58%) of the mothers having had 4 or more children. The majority, 97.5% (n = 2024), of immediate previous pregnancy outcomes were live births (Table 2).

**Table 2:** **Percentage distribution by obstetric history for reproductive women in age group of 15–49, northern Ethiopia, 2009 to 2012**

**Characteristics**	**Categories**	**n**	**%**
**Year of delivery**	Sep 2009-Aug 2010	475	20.1
	Sep 2010-Aug 2011	760	32.2
	Sep 2011-Aug 2012	1,126	47.7
**First pregnancy**	Yes	284	12.0
	No	2,077	88.0
**Current total born**	Singleton	2,317	98.1
	Twin	44	1.9
**Current pregnancy outcome**	Live birth/single	2,313	98.0
	Live birth/twin	44	1.9
	Still birth/abortion	4	0.2
**Birth order**	1	284	12.0
	2-3	711	30.1
	4-6	932	39.5
	7+	434	18.4
**Previous pregnancy outcome**	Live birth	2,024	97.5
	Still birth	32	1.5
	Spontaneous abortion	21	1.0

More than three-quarters, 76.5% (95% *CI: 74.8%-78.2%*) (n = 1806) of respondents had attended an ANC clinic at least once during the study period. However, only 6.4% (n = 116) of these mothers had made four or more ANC visits. Just under three-quarters, 70.0% (n = 1653), of the women had been tested for HIV, and less than one-fifth (18.7%; n = 441) received an anti-tetanus (TT) vaccine during latest pregnancy. Of these, the vast majority, 94.1% (n = 415), received their anti-tetanus vaccine for the first time (TT_1_). During their current pregnancy, 70.5% (n = 1664) of mothers slept under a bed net. Of these, 86.5% (n = 1440) reported that the bed net was treated with insecticide (Table 3).

**Table 3:** **Antenatal care and institutional delivery services utilization of reproductive women in age group of 15–49, northern Ethiopia, 2009 to 2012**

**Characteristics**	**Categories**	**n**	**%**
**Attend ANC**	Yes	1,806	76.5
	No	555	23.5
**Number of ANC visits (n = 1806)**	1	694	38.4
	2	691	38.3
	3	305	16.9
	> = 4	116	6.4
**HIV tested during pregnancy**	Yes	1,653	70.0
	No	708	30.0
**TT vaccine received during current pregnancy (verification by maternal card)**	Yes	441	18.7
	No	1,920	81.3
**Type TT vaccine received during current pregnancy**	TT1	415	94.1
	TT2	305	69.2
	TT3	208	47.2
	TT4	165	37.4
	TT5	125	28.3
**Sleep under bed net**	Yes	1,664	70.5
	No	697	29.5
**Bed net treated (n = 1664)**	Yes	1,440	86.5
	No	183	11.0
	I don’t know	41	2.5
**Place of delivery**	Home	1722	72.9
	Hospital	316	13.4
	Health center	323	13.7
**Skilled delivery**	Yes	662	28.0
	No	1696	72.0
**Who attend the delivery at home? (n = 1722)**	Doctor/nurse/health officer/health assistant	23	1.0
	Health extension worker	172	7.3
	Trained traditional birth attendant	129	5.5
	Untrained traditional birth attendant	1,163	49.3
	Mother herself	74	3.1
	Other	161	6.8

Only 27.1% (95% *CI: 25.3%-28.9%*) (n = 639) of mothers gave birth at a health institution. The majority, 72.9% (n = 1722), of mothers have gave birth at home. Among mothers who gave birth at home, half (49.3%; n = 1163) were attended by Untrained Traditional Birth Attendants (UTBA). About one third (28.0%; n = 662) of the total births were attended by skilled birth attendants (Table 3).

### ANC and institutional delivery services utilization

**Figure 1:**
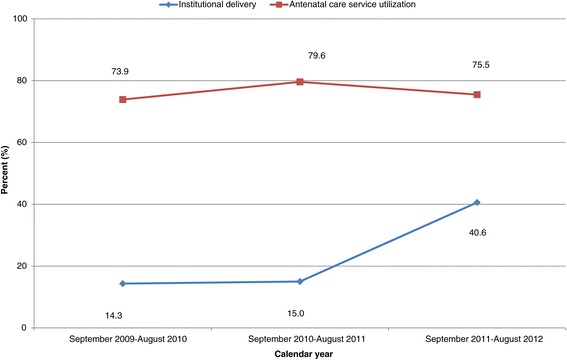
**Pattern of antenatal care services utilization and place of delivery across calendar year in northern Ethiopia between 2009 and 2012 (trend of odds χ** ^**2**^ **: institutional delivery, p < 0.001 and antenatal care, p = 0.9486).**

Institutional delivery service use increased from 14.3% in 2010 to 40.6% in 2012, while ANC service utilization did not change significantly (Figure 1).

**Figure 2:**
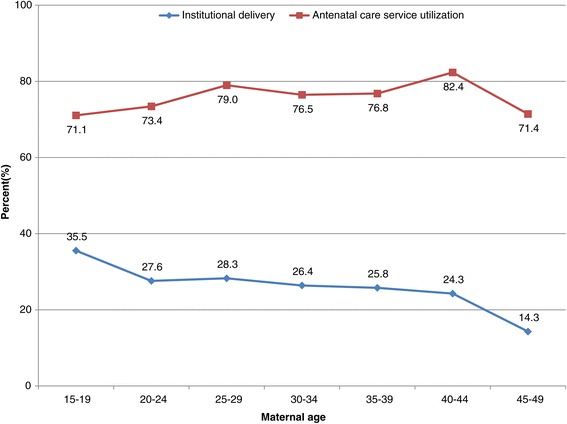
**Pattern of antenatal care and institutional delivery services utilization across age categories in northern Ethiopia between 2009 and 2012 (trend of odds χ** ^**2**^ **: institutional delivery, p = 0.0389 and antenatal care, p = 0.0636).**

More than a third (35.5%) of mothers in the age group 15–19 years gave birth in a health institution, while twice as many mothers in the same age category received ANC services (71.1%). Similarly, the proportion of women aged between 35 and 39 who used ANC services was three times (76.8%) higher than the proportion in the same age group who delivered at health institution (25.8%). In general, the proportion of women who had an ANC visit was 2 to 3 times higher than the proportion of mothers who gave birth at a health institution. As age increased the proportion of women who delivered at health institution tended to decline (Figure 2).

**Figure 3:**
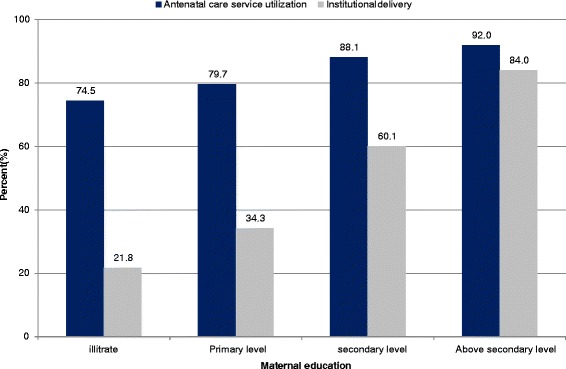
**Pattern of antenatal care and institutional delivery services utilization across maternal educational level in northern Ethiopia Between 2009 and 2012 (trend of odds χ** ^**2**^ **: antenatal care, p < 0.001 and institutional delivery, p < 0.001).**

Three-fourths (74.5%) of illiterate mothers had made an ANC visit, whereas less than a quarter (21.8%) of them had an institutional delivery. By contrast, 88.1% of mothers with secondary education had made an ANC visit, with three-fifths (60.1%) of these mothers having an institutional delivery. Overall, as educational level increased, the proportions of mothers who attended ANC and gave birth at a health institution also tended to increase (Figure 3).

**Figure 4:**
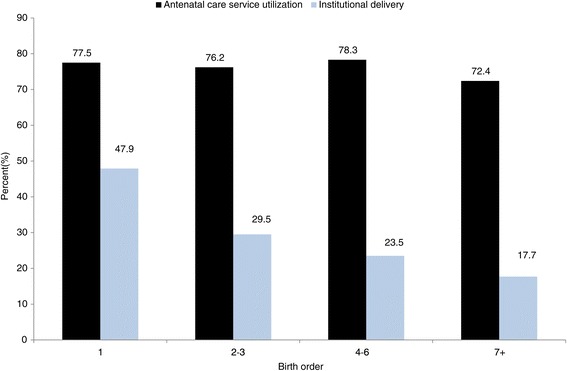
**Pattern of antenatal care and institutional delivery services utilization across maternal birth order in northern Ethiopia, between 2009 and 2012 (trend of odds χ** ^**2**^ **: antenatal care, p = 0.2515 and institutional delivery, p < 0.001).**

Although more than three quarters (77.5%) of mothers made an ANC visit during their first pregnancy, less than half (47.9%) of them gave birth at a health institution. The proportions of mothers with 4 to 6 children who attended ANC and who had an institutional delivery were 78.3% and 23.5%, respectively. The data indicate that the proportion of mothers who give birth at a health institution decreases as their parity increases, even if ANC attendance does not change significantly (Figure 4).

**Figure 5:**
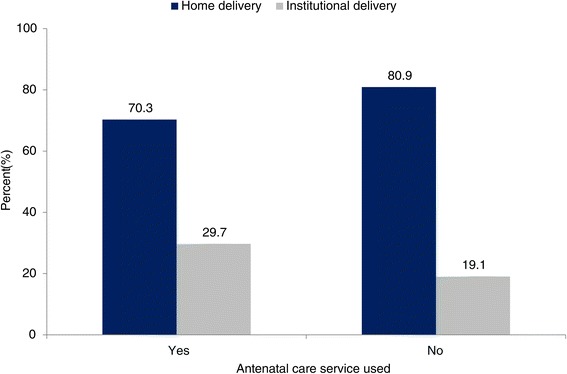
**Antenatal care service utilization and place of delivery in northern Ethiopia between 2009 and 2012 (Pearson χ** ^**2**^ **, p < 0.001).**

The overall proportions of mothers who delivered at a health institution were 29.7% and 19.1%, respectively, among mothers who had attended an ANC clinic and those who had not (Figure 5).

### Determinants of ANC service utilization

In bivariate analysis, maternal age, residence, maternal educational level, marital status of mother and maternal occupation were found to be significantly associated with ANC service utilization. In multivariate logistic regression analysis, the age of the mother, residence, maternal educational status, and maternal occupation were found to be significantly associated with ANC service utilization (Table 4).

**Table 4:** **Determinants of antenatal care service utilization of reproductive women in age group of 15–49, northern Ethiopia, 2009 to 2012**

		**Antenatal care attended**				
**Characteristics**	**Categories**	**Yes**	**No**	**COR (95% CI)**	**AOR (95% CI)**	**β**
**Age (Years)**	15-19	86	35	1.00	1.00	
	20-24	362	131	1.12 (0.72,1.75)	1.45 (0.90,2.36)	0.373
	25-29	447	119	1.53 (0.98,2.38)	2.15 (1.29,3.58)*	0.764
	30-34	481	148	1.32 (0.86,2.04)	2.02 (1.21,3.37)*	0.703
	35-39	298	90	1.35 (0.85,2.13)	2.17 (1.26,3.72)*	0.773
	40-49	132	32	1.68 (0.97,2.91)	2.84 (1.52,5.28)*	1.149
**Residence**	Urban	151	17	2.89 (1.73, 4.81)*	2.20 (1.25,3.87)*	0.783
	Rural	1655	538	1.00	1.00	
**Maternal educational status**	No formal education	1297	444	1.00	1.00	
	Primary education	360	92	1.34 (1.04, 1.73)*	1.47 (1.10,1.95)*	0.382
	Secondary education and above	149	19	2.68 (1.65,4.38)*	2.38 (1.36,4.18)*	0.858
**Marital status**	Single	260	59	1.00	1.00	
	Married	1429	478	0.68 (0.50,0.92)*	1.45 (0.87,2.42)	−0.374
	Dissolved	117	18	1.48 (0.83,2.61)	1.45 (0.78,2.68)	−0.002
**Maternal occupation**	Farmer	154	21	1.60 (0.89,2.88)	2.31 (1.23,4.35)*	0.835
	Daily laborer	108	26	0.91 (0.52,1.60)	1.36 (0.74,2.51)	0.31
	Housewife	1272	446	0.62 (0.43,0.91)*	1.09 (0.66,1.78)	0.088
	Student	112	27	0.91 (0.51,1.58)	1.05 (0.55,2.02)	0.054
	Other	160	35	1.00	1.00	1

COR-Crude Odds Ratio, AOR-Adjusted Odds Ratio (adjusted for all other variables in the table), CI-Confidence Interval (95%), *statistically significant at P = 0.05, all p-values are produced using Z test.

Mothers in the age categories of 25–29, 30–34 and 35–39 years were slightly more than twice as likely to attend ANC than mothers aged 15–19, (AOR = 2.15, 95% CI = [1.29,3.58]; AOR = 2.02, 95% CI = [1.21,3.37]; and AOR = 2.17, 95% CI = [1.26,3.72], respectively), while mothers aged 40–49 years were nearly three times more likely to attend ANC than mothers aged 15–19 years (AOR = 2.84, 95% CI = [1.52, 5.28]).Urban residents were more than twice as likely as rural dwellers to use ANC services (AOR = 2.20, 95% CI = [1.25,3.87]).ANC service utilization also increased with the level of women’s education. The odds of receiving ANC at least once among women who had some education were significantly higher than they were for women with no formal education (AOR = 1.47, 95% CI = [1.10, 1.95] for those with primary education; and AOR = 2.38, 95% CI = [1.36, 4.18] for those with secondary education and above) (Table 4).

### Determinants of institutional delivery service utilization

In the bivariate analysis, maternal age, residence, ethnicity, religion, maternal education, marital status of mother, maternal occupation, ANC attendance, and maternal birth order were found to be significantly associated with institutional delivery. However, in multivariate logistic regression, maternal age, residence, maternal educational status, ANC attendance, and birth order were found to be significantly associated with the institutional delivery service utilization (Table 5).

**Table 5:** **Determinants of institutional delivery of reproductive women in age group of 15–49, northern Ethiopia, 2009 to 2012**

		**Institutional delivery**				
**Characteristics**	**Categories**	**Yes**	**No**	**COR (95% CI)**	**AOR (95% CI)**	**β**
**Age (Years)**	15-19	43	78	1.00	1.00	
	20-24	136	357	0.69 (0.45,1.05)	1.29 (0.77,2.15)	0.254
	25-29	160	406	0.71 (0.47,1.08)	1.86 (1.07,3.25)*	0.623
	30-34	166	463	0.65 (0.43,0.98)*	2.36 (1.32,4.22)*	0.858
	35-39	100	288	0.63 (0.41,0.97)*	3.05 (1.62,5.71)*	1.114
	40-49	37	127	0.53 (0.31,0.89)*	3.24 (1.58,6.64)*	1.242
**Residence**	Urban	154	14	38.43 (22.04,67.03)*	27.73 (15.09,50.98)**	3.321
	Rural	488	1705	1.00	1.00	
**Ethnicity**	Tigray	633	1714	1.00	1.00	
	Other	9	5	4.87 (1.63,14.60)*	1.72 (0.40,7.41)	0.534
**Religion**	Orthodox	630	1712	1.00	1.00	
	Other	12	7	4.66 (1.83,11.89)*	0.88 (0.23,3.35)	−0.129
**Maternal educational status**	No formal education	380	1361	1.00	1.00	
	Primary education	155	297	1.87 (1.49,2.34)*	1.24 (0.93,1.66)	0.217
	Secondary education and above	107	61	6.28 (4.50,8.78)*	1.72 (1.07,2.79)*	0.541
**Marital status**	Single	146	173	1.19 (0.79,1.79)	0.88 (0.53,1.47)	0.427
	Married	440	1467	0.42 (0.30,0.61)*	1.54 (0.91,2.59)	−0.129
	Dissolved	56	79	1.00	1.00	
**Maternal occupation**	Farmer	61	114	0.42 (0.28,0.64)*	1.49 (0.86,2.56)	0.393
	Daily laborer	51	83	0.49 (0.31,0.76)*	1.22 (0.69,2.14)	0.198
	Housewife	350	1368	0.20 (0.15,0.27)*	0.89 (0.54,1.47)	−0.116
	Student	71	68	0.82 (0.53,1.28)	1.32 (0.74,2.35)	0.281
	other	109	86	1.00	1.00	
**Attend ANC**	Yes	536	1270	1.79 (1.41,2.26)*	1.39 (1.07,1.79)*	0.325
	No	106	449	1.00	1.00	
**Birth order**	1	136	148	4.26 (3.04,5.98)*	3.01 (1.69,5.35)**	1.101
	2-3	210	501	1.94 (1.45,2.61)*	1.85 (1.20,2.84)*	0.615
	4-6	219	713	1.42 (1.07,1.90)*	1.41 (1.00,1.98)*	0.345
	7^+^	77	357	1.00	1.00	

COR-Crude Odds Ratio, AOR-Adjusted Odds Ratio (adjusted for all other variables in the table), CI-Confidence Interval (95%), *statistically significant at P = 0.05, **statistically significant at P < 0.001, all p-values are produced using Z test.

Mothers aged 25 years and above were two to three times more likely to give birth in a health facility than mothers aged 15–19 years. Mothers who were urban residents were 28 times more likely (AOR = 27.73, 95% CI = [15.09, 50.98]) to give birth in a health facility than rural mothers. Mothers with secondary education and above had almost twice the odds of giving birth in a health facility than illiterate mothers (AOR = 1.72, 95% CI = [1.07, 2.79]). Making at least one ANC visit during pregnancy was also found to be an independent predictor of institutional delivery service utilization (AOR = 1.39, 95% CI = [1.07, 1.79]). Similarly, mothers with parity of one had three times the likelihood of delivering at a health facility than mothers with high parity (seven children and above) (AOR = 3.01, 95% CI = [1.69, 5.35] (Table 5).

## Discussion

Our analysis of community-based, longitudinal data from Tigray region, Ethiopia, has attempted to identify the magnitude and determinants of ANC and institutional delivery services utilization. This Discussion focuses first on the rates of utilization of the two types of service before turning to the determinants of utilization.

### Antenatal care service utilization

The results showed that more than three-quarters (76.5%) of pregnant women in the KA-HDSS study area had made at least one ANC visit. This is much higher than the national average, estimated in two different studies from 2011 and 2014 respectively, at 34% and 40% [11,24]. Some of this large difference could be explained by the methodological differences used by the different studies. The use of longitudinal data, as in our study, can decrease recall bias during data collection, potentially giving a more accurate, and in this case, higher, estimate. However, we believe that the major reason for the difference is the substantially increased efforts made by the Tigray Regional Health Bureau to expand maternal health care services after the 2011 EDHS reported continued and unacceptably high levels of maternal mortality [24].

In spite of this policy success, however, we found that only 6.4% of mothers had made four or more ANC visits, as recommended by WHO. This figure is lower than those found in other studies conducted in Ethiopia (19% [24], 48% [30] and 55% [32]), as well as in several other low and middle-income countries: Sudan (11%) [12], Democratic Republic of Congo (53%) [33], Pakistan (35%) [34] and Indonesia (78%) [18]. These differences could be at least partially explained by socio-demographic, economic and cultural variations between the population groups under investigation in these studies, factors which can affect utilization of maternal health care services [17,35]. Population variables like these are not, of course, easily addressed by the health services, but health workers can, and should, counsel and encourage all pregnant women during their first ANC visit, to ensure that they continue their pregnancy follow up at least four times as per WHO’s recommendation [29].

### Place of delivery and birth attendants

Our study found that 27% of mothers gave birth at a health institution, which is a higher proportion than has been reported elsewhere in Ethiopia (10% [24], 15% [11], 12% [13], 5% [30], and 12% [32]). This difference could be partially explained by the fact that mothers in our study had better educational status and better ANC service utilization compared to the mothers in these other studies, since these factors are known to have an impact on institutional delivery service utilization. In addition, as part of the efforts made by Tigray Regional Health Bureau to decrease maternal mortality, expanding utilization of institutional delivery services has been a major focus area for policy makers. Further, the continuous monitoring of health data by the HDSS in the study area could have acted as a health promoting intervention in its own right, with the result that maternal health service uptake could have increased.

That said, institutional delivery in these Ethiopian studies is still much lower than has been found by research conducted in a number of other countries, including Democratic Republic Congo (94%) [33], Malawi (50%) [36], Zambia (34%) [36], Indonesia (63%) [18], India (84%) [22], and Pakistan (41%) [34]. Much of the difference may be due to the non-Ethiopian studies being conducted in urban and peri-urban areas, where institutional delivery is more common. Nonetheless, it would appear that both Tigray region and Ethiopia as a whole have relatively low levels of institutional delivery, and efforts must be made to understand the reasons for this, and then address it at policy level.

Half of (49.3%) the home deliveries in our study were attended by Untrained Traditional Birth Attendants (UTBA), a higher rate than reported by other Ethiopian studies, where between 3% and 38% of home deliveries were attended by UTBAs [13,24,30,32]. A previous study in our study area found that women very much appreciate the services of TBAs, who are perceived as being caring of the women whom they deliver at home [35]. Since they are accepted by mothers, and they are close to the community, TBAs could be trained to educate mothers on importance of institutional delivery and serve as referring agents to health facilities when labour starts.

Of all births, 28.0% were attended by a skilled birth attendant, which is not dissimilar to another study conducted in Tigray region in 2009, where 25% of births were attended by a health professional or a trained Traditional Birth Attendant [30]. Higher levels of skilled birth attendance were reported in a study in Kenya (49.6%) [15], which could be explained by differing cultural norms and relative inaccessibility of health institutions in our study area as compared to the Kenyan study.

The finding that the use of ANC services is higher than the use of institutional delivery services is consistent with the results of several previous studies from Ethiopia [11,13,24,30,32] as well as one conducted in Nigeria [17]. One of the reasons given to explain this is the unpredictable nature of the onset of labour combined with the difficulty in accessing health facilities in an emergency in resource-poor environments. Poor road networks, limited transportation means, and an under-served population in terms of health facilities are likely to contribute to this problem [17]. It is therefore important to give adequate counseling for mothers on birth preparedness and planning for safe delivery during their ANC visits.

### Determinants of maternal health care service utilization

Our findings indicated that as the age of mothers increased, they were more likely to attend ANC, a finding consistent with that of a systematic review conducted in low and middle-income countries [16]. The risk of pregnancy-related complications is higher in very young mothers, and further, the fact that they have less experience of pregnancy than older mothers could make them less aware of the symptoms of complications and danger signs during pregnancy. Thus, ANC services should be promoted in particular for younger mothers in order to improve maternal health outcomes in Tigray region.

An almost opposite finding emerged with regard to institutional delivery, whereby mothers having their first child (who would generally be younger) were three times more likely to deliver at a health facility than mothers who had had seven or more (who would generally be older). Similar results were reported from a study conducted in Uganda [37], the likely explanation being that mothers may be more confident about the birth due to their experience of previous deliveries, which may reduce their maternal health-seeking behavior. Tailored counseling during pregnancy on the benefits of institutional delivery and risks of home delivery could benefit these older, higher parity women.

Urban mothers were twice as likely to make at least one ANC visit as compared to their rural counterparts, and 28 times more likely to give birth in a health facility. Better information about and access to services are likely to explain much of these differences. Further, since urban women have generally received more education than rural women, educational attainment levels probably also played a role here: mothers with secondary education and above were between three and four times more likely to give birth in a health facility than illiterate mothers.

Our findings about the associations between place of residence and educational level, and utilization of maternal health services, were expected, since they are broadly consistent with a swathe of other studies from Ethiopia [13,19,32], elsewhere in Africa [12,13,15,17,37,38], and in some Asian settings [18,20,39,40]. They point to two broad areas of intervention, namely the need to increase access to maternal health services in rural areas, and promoting education for girls. The Tigray Regional Health Bureau has substantially increased the number of ambulances in the last two or three years, particularly in rural areas, so we expect that institutional deliveries will have increased as a result. The current findings may therefore be used as a baseline for evaluating the effect of these improved ambulance services on institutional delivery rates in the region. With regards to education for girls, the situation continues to improve in Tigray region: the proportion of females aged 6 years and over with no education fell from 77% in 2000 to 46% in 2014 [11,24,41]. However, continued efforts are clearly still needed here in order to ensure that all girls receive an education.

### Limitations

Despite the overall methodological strength of this study, which has been based on longitudinal, community-based data, some variables such as accessibility of health services, social and economic status, and husband/partner characteristics were not included. Furthermore, social factors like decision-making with partners, which could have important implication in maternal service utilization [42], were not included as part of the surveillance process. Factors related to quality of maternal health services like waiting time and qualification of service providers were also not included as predictor variables in the study. Finally, it is important to note that health service utilization in the surveillance area, including maternal health care, could be increased due to the awareness raised in the community by the regular visits of the KA-HDSS team to collect data.

## Conclusions

Despite low levels of institutional delivery service utilization, a relatively high proportion of mothers attended antenatal care services at least once. However, the proportion of mothers who made the WHO recommendation of four ANC visits was very low. Thus, increased efforts should be made to increase the number of ANC visits made by pregnant women, which could be accomplished through counseling of mothers at their first ANC contact.

The large gap between the proportion of women who received at least some ANC and the proportion of women who had an institutional delivery needs to be reduced. Since we found that the women most likely to deliver in a health facility were younger, educated, had attended ANC, and had fewer children, it may be important for programs and policy to make special efforts to address the needs of older, less educated women with more children. Tailored counseling should be provided for these women during ANC on the importance of institutional delivery and on the risks associated with home delivery. Consideration should also be given to promoting institutional delivery specifically for women who do not come for ANC, for example by training health extension workers who live in the community to inform pregnant women about the benefits of giving birth in a health facility, along with discussion about how they could make plans in advance of labour in order to facilitate this.

The findings from this quantitative study could be further explained, and additional means of increasing utilization of maternal health services in Tigray region could be identified, by qualitative investigations into the relevant social, cultural and economic factors. We hope to pursue such studies in future.
